# Process–Structure Co-Optimization of Glass Fiber-Reinforced Polymer Automotive Front-End Module

**DOI:** 10.3390/ma18133121

**Published:** 2025-07-01

**Authors:** Ziming Chen, Pengcheng Guo, Longjian Tan, Tuo Ye, Luoxing Li

**Affiliations:** 1 College of Mechanical and Vehicle Engineering, Hunan University, Changsha 410082, China; 2 Intelligent Manufacturing and Mechanical Engineering, Hunan Institute of Technology, Hengyang 421002, China; 3 Research Institute of Hunan University in Chongqing, Hunan University, Chongqing 400044, China

**Keywords:** glass fiber reinforced polymer, front-end module, finite element method, topology optimization, injection molding, process parameters optimization, injection molding history mapping

## Abstract

For automotive GFRP structural components, beyond structural design, the warpage, residual stress/strain, and fiber orientation inevitably induced during the injection molding process significantly compromise their service performance. These factors also diminish the reliability of performance assessments. Thus, it is imperative to develop a process–structure co-optimization approach for GFRP components. In this paper, the performance of a front-end module is evaluated through topological structure design, injection molding process optimization, and simulation with mapped injection molding history, followed by experimental validation and analysis. Under ±1000 N loading, the initial design shows excessive displacement at the latch mounting points (2.254 mm vs. <2.0 mm limit), which is reduced to 1.609 mm after topology optimization. By employing a sequential valve control system, the controls of the melt line and fiber orientation are is superior to thatose of conventional gating systems. The optimal process parameter combination is determined through orthogonal experiments, reducing the warpage to 1.498 mm with a 41.5% reduction compared to the average warpage of the orthogonal tests. The simulation results incorporating injection molding data mapping (fiber orientation, residual stress–strain) show closer agreement with experimental measurements. When the measured displacement exceeded 0.65 mm, the average relative error $E_{r}$, range R, and variance $s^{2}$ between the experimental results and mapped simulations were 11.78%, 14%, and 0.002462, respectively, validating the engineering applicability of this method. The methodology and workflow can provide methodological support for the design and performance assessment of GFRP automotive body structures, which enhances structural rigidity, improves control over injection molding process defects, and elevates the reliability of performance evaluation.

## 1. Introduction

Against the backdrop of accelerated global carbon neutrality strategies, lightweighting has become one of the core technologies driving the green transformation of the automotive industry [1,2,3]. Glass fiber-reinforced polymer (GFRP), as a representative lightweight material, demonstrates particularly outstanding performance advantages: optimizing the fiber type and distribution can achieve strength comparable to steel while maintaining a density only one-fourth that of steel [4,5]. These advantages enable GFRP to achieve a 30–50% weight reduction while maintaining structural integrity [6,7]. Industry practices have shown that in critical component applications such as Tesla Model S body components and BMW i-series chassis parts, GFRP has exhibited an exceptional weight-reduction performance, exceeding 40% compared to traditional materials [8,9]. However, the anisotropic material behavior induced by fiber orientation in GFRP leads to errors of 15–20% when isotropic simulation models are employed [10], severely constraining the accurate evaluation of product performance.

Common manufacturing methods for GFRP structures include injection molding, compression molding, and additive manufacturing [11,12,13]. Among these, injection molding is a closed-mold process that involves injecting a high-temperature mixture of polymer pellets and short fibers into a mold cavity, offering significant advantages for mass-producing components with complex geometries. Warpage is one of the most common defects in GFRP body structures, which is primarily caused by non-uniform shrinkage and residual stress relaxation [14]. Utilizing the finite element method to simulate injection molding processes enables accurate prediction of warpage deformation, thereby greatly aiding in controlling warpage [15,16,17]. Optimizing the reinforcement rib layout and gate position, as well as adjusting the injection process, can significantly reduce warpage [18,19]. Purgleitner et al. [20] revealed that mold temperature exhibited a positive correlation with mechanical properties, while materials with a lower melt flow rate demonstrated higher susceptibility to weld line effects. The performance of components was predominantly governed by material properties, while process optimization could offer limited performance enhancement. Li et al. [21] conducted a regression analysis based on a rapid heating and cooling system, revealing that warpage is affected by resin temperature in the case of a resin containing 30% glass fibers added to polyamide 66. Bian [22] performed a comparative analysis of six critical parameters including injection velocity, injection pressure, injection temperature, mold temperature, short carbon fiber (SCF) content, and cooling time, revealing that SCF content and injection velocity predominantly influence the warpage. Kumar et al. [23] employed a Taguchi L27 orthogonal array combined with grey relational analysis to optimize the injection molding process for cam sleeves, achieving a significant improvement in product quality. Park et al. [24] established a process–property correlation model for injection molding and developed a parameter deviation compensation algorithm to effectively control quality defects. Kitayama et al. [25] conducted multi-objective optimization of the injection molding process using a radial basis function optimization algorithm with warpage and cycle time as the objectives, which effectively reduced warpage and shortened the cycle time. Additionally, the combined application of variable-pressure filling (low → high pressure) and rapid heat cycle molding technology could also effectively enhance the quality and efficiency simultaneously [26].

Tang et al. [27] found that the composite of polypropylene and 30% glass fiber-reinforced polypropylene achieved the highest bonding strength, and its best injection molding process was obtained through response surface methodology optimization, with a tensile strength of 28.3 MPa. Chauhan et al. [28] optimized critical parameters such as the melting temperature and injection pressure by integrating a Taguchi experimental design with Moldflow simulation. The optimal process to control warpage was obtained through analysis of variance and signal-to-noise ratio evaluation. In recent years, advanced models have been applied to the prediction and optimization of defects resulting from injection molding processes [29]. Öktem et al. [30] developed a systematic approach combining a uniform experimental design, Kriging modeling, and multi-objective optimization, resulting in a 7.7% reduction in wheel hub warpage and 11.91% improvement in ejection time. Mukras et al. [31] established a Kriging model using injection molding processes as design variables, with warpage and volumetric shrinkage rate as evaluation indicators. After optimization using a genetic algorithm, the molding defects were reduced by approximately 7%. Yang et al. [32] optimized the injection molding process for automotive brackets through regression analysis and genetic algorithm–back propagation (GA-BP) neural network prediction, achieving a reduced volumetric shrinkage rate of 13% and decreasing warpage to 0.706 mm. Liu et al. [33] integrated adaptive-boosting with a GA-BP neural network, further enhancing the stability and accuracy of warpage deformation prediction. Guo et al. [34] combined the particle swarm optimization–BP model with the genetic algorithm to optimize the process parameters for microcellular foaming injection molding, achieving a warpage reduction to 0.7038. Dogossy et al. [35] proposed a multi-stage simulation approach for thick-walled injection-molded composites integrating different finite element software.

Fonseca et al. [36] employed topology optimization and free-size optimization to determine the optimal rib configuration and positioning of metal inserts for a recycled carbon fiber-reinforced plastic/metal hybrid engine mount, which resulted in a 36% weight reduction compared to the steel benchmark model. Gao et al. [37] developed an integrated simulation approach based on Moldex3D and Abaqus for predicting the mechanical properties of injection-molded fiber-reinforced polymers, which were validated with experimental results from the existing literature. Kim et al. [38] manufactured a carbon–epoxy composite automotive lower arm using a micro-genetic algorithm for stacking sequence optimization. The optimized composite arm achieved a 50% weight reduction compared to its steel counterpart while doubling both stiffness and buckling strength. Park et al. [39] proposed a three-stage optimization framework (topology optimization—injection molding coupling analysis—multi-objective optimization) for short fiber composite automotive front body design to achieve an optimal balance between material anisotropy and lightweight characteristics on the Pareto frontier. Smith et al. [40] confirmed the necessity of considering anisotropy in composite design through comparing simulations with experiments, revealing that isotropic assumptions would lead to a significant performance evaluation deviation.

**Figure 1:**
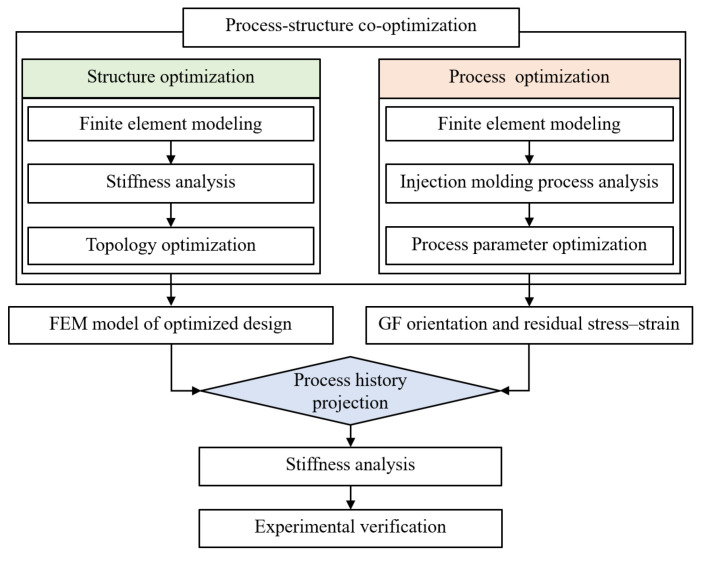
Flowchart of process–structure co-optimization methodology.

As mentioned above, apart from the structural design, the warpage, residual stress–strain, and fiber orientation induced by injection molding significantly could significantly affect the structural performance, and these factors also impact the accuracy of performance evaluations. Therefore, it is essential to establish a process–structure co-optimization approach for GFRP components. This study focuses on a GFRP front-end module, where structural optimization is employed to enhance the stiffness of key mounting points, followed by injection molding process optimization through injection molding simulation based on an orthogonal experiment, and service performance evaluation by mapping injection molding history to structural mechanics simulation. The flowchart of the methodology proposed is illustrated in Figure 1, which can provide technical support for the design and performance evaluation of GFRP automotive body structures.

## 2. Stiffness Simulation of Mounting Points and Structure Optimization

### 2.1. Finite Element Modeling

**Figure 2:**
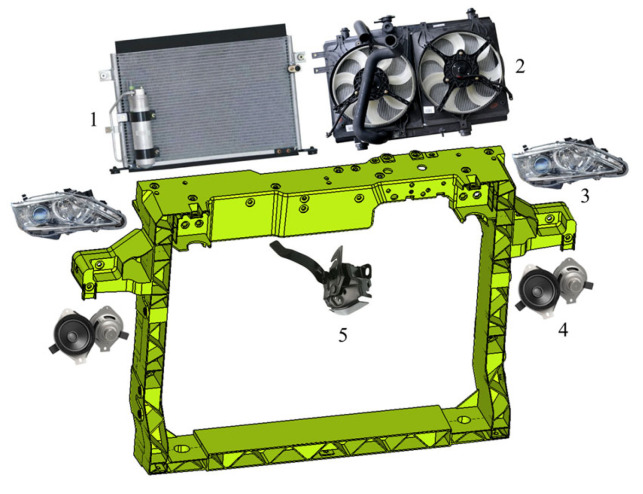
Structure of the GFRP front-end module and its ancillary components: (1) condenser; (2) radiator; (3) headlight; (4) horn; (5) latch.

The front-end module mainly integrates components such as hood latch, headlight, radiator, and horn, as shown in Figure 2. These mounting points need to possess sufficient stiffness to withstand road bumps, interference induced by shaking, abnormal vibration noise, and other issues, with specific stiffness indexes listed in Table 1. In-mold assembly molding process is adopted for the GFRP front-end module, which involves precisely positioning pre-stamped metal parts inside its injection molds, using high-pressure injection molding to encapsulate the metal inserts with molten plastic. After cooling and solidification, a metal–plastic integrated structure is formed. Compared to traditional stamping-welding and pure injection molding, this method not only delivers higher dimensional stability and superior mechanical performance but also achieves a weight reduction of over 40% compared to traditional steel components [41,42].

**Figure 3:**
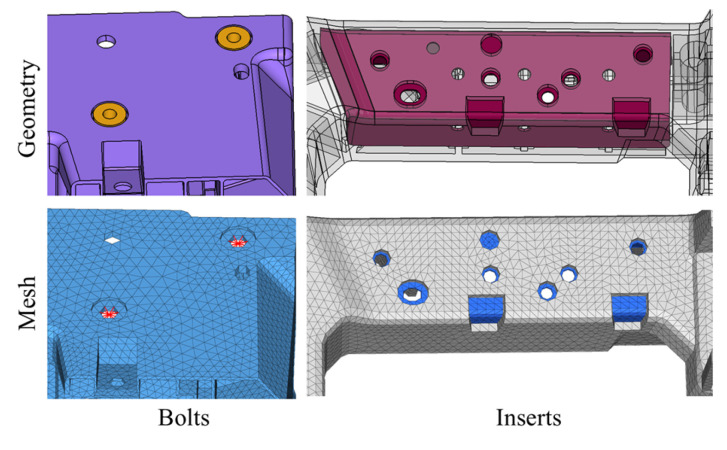
Schematic of the treatment of bolts and inserts.

Before meshing, geometric preprocessing is required to ensure mesh quality. First, import the CATIA model into Hypermesh 2022.1 and remove fillets and steps smaller than 0.3 mm. Then, perform meshing using C3D10 tetrahedral elements to accurately represent thickness variation and complex geometry while preventing over-stiffness in the simulation. The bolts and inserts are molded into the front-end module via in-mold injection, exhibiting elastic modulus two orders higher than that of GFRP. For finite element modeling, the bolt holes are treated with coupling constraints, while all contact surfaces between the inserts and the GFRP body employ shared-node meshing, as shown in Figure 3. A global mesh size of 5 mm is adopted, resulting in a total of 327,466 elements with minimum element size of 0.8 mm. The GFRP front-end module utilizes PP + LGF40 material, which is characterized by the addition of 40% long glass fiber in PP material [43]. GFRP is conventionally treated as isotropic, requiring only three fundamental parameters for stiffness simulation: elastic modulus (5240 MPa), Poisson’s ratio (0.34), and density (1.22 × 10^−9^ ton/mm^3^).

### 2.2. Analysis of Mounting Point Stiffness

**Figure 4:**
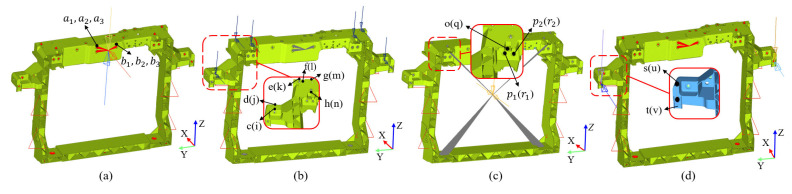
Constraint and loading schematics for mounting point stiffness simulation and measurement: (**a**) latch mounting points; (**b**) headlight mounting points; (**c**) radiator mounting points; (**d**) horn mounting points.

A 1000 N load is applied in the vehicle’s +X and ±Z directions on the latch mounting points. The right-side measurement points include $a_{1}$ (+X), $a_{2}$(+Z) and $a_{3}$ (−Z), with left-side symmetrical points $b_{1}$ (+X), $b_{2}$(+Z) and $b_{3}$ (−Z), as illustrated in Figure 4a. A 100 N load is applied along the +X and −Z directions on the headlight mounting points. The right-side measurement points are d/e/g (+X) and c/f/h (−Z), with symmetrical left-side points j/k/m (+X) and i/l/n (−Z), as shown in Figure 4b. Due to the extremely high elastic modulus of aluminum alloy (70 GPa) as compared to GFRP, the mounting points of the aluminum alloy radiator are rigidly coupled to its centroid using coupling constraints, followed by loading of 600 N in the ±X and −Z directions. The measurement points include o (−Z) and $p_{1}$/$p_{2}$ (+X) on the right side, with symmetrical points q (−Z) and $r_{1}$/$r_{2}$ (+X) on the left side, as depicted in Figure 4c. For the horn mounting points, a 300 N load is applied along the +X and −Z directions. The right-side measurement points are s (−Z) and t (+X), with corresponding left-side points u (−Z) and v (+X), as shown in Figure 4d.

The constraint and loading for stiffness analysis are based on experimental test specifications, as shown in Figure 4. In finite element analysis models, load cases and analysis steps are established according to the loading conditions of each mounting point. The analysis step type is specified as static, linear perturbation. After configuring the step parameters and defining the field variable outputs, these models are submitted to the solver of Abaqus 2020. Table 2 presents the displacement response statistics of each point of the initial design. According to Table 1, the displacement response at the latch measurement point exceeds regulatory limits, while all other measurement points meet the design requirements. This indicates insufficient stiffness for the latch mounting points, necessitating structural optimization.

### 2.3. Structure Optimization

**Figure 5:**
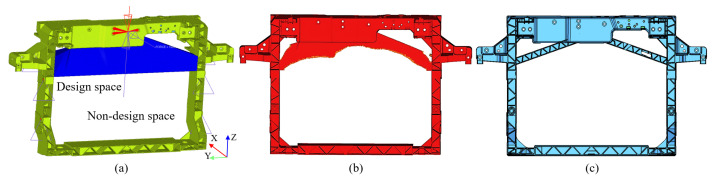
Structural optimization design models: (**a**) topology optimization model; (**b**) topology optimization result; (**c**) optimized design.

To address the insufficient stiffness of the latch mounting points in the ±Z direction, adding support structures to its upper beam can effectively enhance structural stiffness. However, key parameters such as the cross-section, geometric dimension, and spatial location are difficult to determine empirically. Topology optimization is a numerical optimization method that seeks optimal material distribution within a defined design domain based on given loading conditions, constraints, and performance objectives, enabling the rapid derivation of a structure and layout that meet the engineering requirements [44,45]. Prior to finalizing the optimized layout, a spatial interference check must first be conducted to ensure assembly compatibility with surrounding components. Considering the matching relationship between the front-end module and the radiator, along with piping layout requirements, the region illustrated in Figure 5a is ultimately selected as the design space for topology optimization. Topology optimization is performed using the displacement at points a and b under 1000 N in the ±Z directions, along with the optimized region volume, as design responses. Displacement constraints (a, b < 2 mm) are imposed with volume minimization as the objective. The optimization result (Figure 4b) clearly shows the outline for support structure design. Based on this and considering manufacturing feasibility, two inclined support structures are designed on both sides of the latch with the following parameters: 30 mm width, 15 mm height, 2 mm rib thickness, and 3° draft angle. The optimized front-end module structure is shown in Figure 5c.

The deformation displacement of the topology-optimized design is listed in Table 2, which indicates that all the points meet displacement requirements under various load cases, including the latch mounting points. In other words, adding two inclined reinforcements to the lower end surface of the upper beam can effectively enhance the latch mounting point stiffness, achieving the given specified performance target.

## 3. Design and Optimization of Injection Molding Process

### 3.1. Design of Injection Molding Process

**Figure 6:**
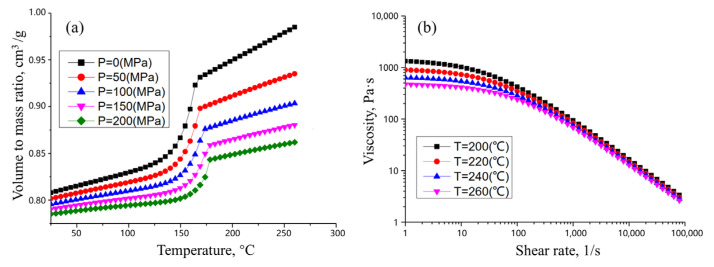
PVT curves of PP + LGF40 GFRP under varying injection pressures (**a**) and viscosity–shear rate curves at different temperatures (**b**).

The service performance of PP + LGF40 GFRP front-end modules depends not only on material and structural design, but also critically on the injection molding process. The basic mechanical properties of PP + LGF40 GFRP are shown in Table 3. It is well known that the PVT (pressure-specific volume–temperature) curve characterizes the variation of volume-to-mass ratio (VMR) under different temperature and pressure conditions, serving as a fundamental basis for determining injection molding process parameters, such as melt temperature, mold temperature, and injection pressure. In addition, from injection to packing and cooling, components inevitably undergo dynamic changes in temperature and pressure, and their final shrinkage behavior is entirely governed by the PVT characteristics. As shown in Figure 6a, PP + LGF40 GFRP exhibits a distinct transition point from solid–liquid coexistence to pure liquid under various pressures, while demonstrating a monotonically decreased VMR with increasing pressure. For GFRP, its viscosity characterizes intermolecular friction, which exhibits distinct non-Newtonian fluid behavior with a nonlinear shear stress–shear rate relationship. The viscosity curve of PP + LGF40 GFRP is shown in Figure 6b, demonstrating a dual dependence on both shear rate and temperature: (1) under isothermal conditions, the viscosity displays a gradual decrease with the increase in shear rate; (2) at fixed shear rates, the viscosity shows a monotonic reduction with temperature elevation.

**Figure 7:**
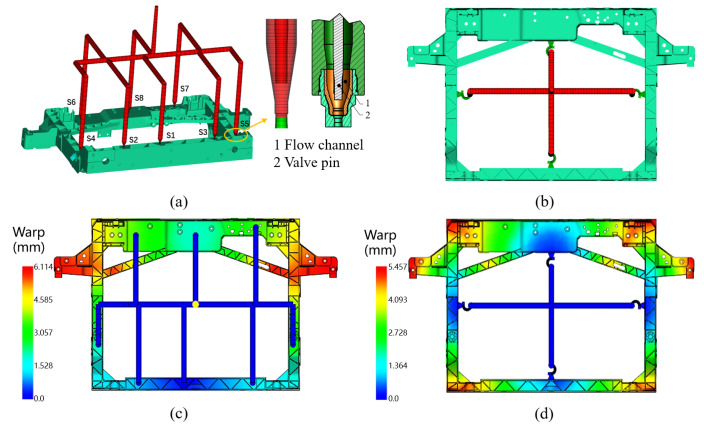
Simulation models and warpage distribution results: (**a**,**c**) sequential valve injection; (**b**,**d**) conventional simultaneous injection.

Rational gating system design can significantly improve injection molding quality, including ensuring complete cavity filling, optimizing fiber orientation, reducing injection pressure, controlling weld line distribution, minimizing warpage, and shortening cycle time [46,47]. According to the structural characteristics of the studied front-end module, the impact of sequential valve injection versus conventional simultaneous injection on molding quality is analyzed. Two models for the injection molding process are established in Moldflow 2020. Case 1 adopts a hot runner system with eight φ5 mm sequential valves (Figure 7a), controlling the melt flow path and filling direction by opening multiple hot runner valves in a specific sequence. Time-controlled filling (S1-S2-S3-S8-S4-S5-S6-S7) is achieved with a total injection time of 6.1 s (Figure 7c). Among them, valve S8 opens at 2 s, and the melt ultimately converges at its left and right hanging ears. Case 2 uses four runners for simultaneous filling (Figure 7b), reducing the total injection time to 5.5 s (Figure 7d), with the melt converging at four corners. Compared to case 1, this case offers a shorter molding cycle.

The weld line is a mechanically weak zone formed by the convergence of two molten fronts in injection molding, with the strength being only 10–90% of its base material, especially for glass fiber-reinforced materials [48,49]. Weld line analysis can predict its location and impact. For annular components, such as front-end modules, the weld line should be controlled in low-stress areas or structurally reinforced regions. Additionally, glass fiber orientation in the PP matrix is determined by melt flow, which can also be predicted through flow analysis [50]. Furthermore, the gating system should ensure balanced gate distribution to avoid defects such as flash and warpage caused by localized high pressure, while maintaining a reasonable injection pressure distribution.

To accurately capture fiber orientation information in injection molding, the mesh is refined with an average element size of 4.5 mm and a minimum element size of 0.5 mm. Based on the default parameters recommended by Moldflow software for PP + LGF40 GFRP, the mold temperature and melt temperature are set to 20°C and 210°C, respectively. Additionally, the packing time is set to 10 s, with packing pressure at 80% of the injection pressure. The front-end module is cooled naturally in the mold, with a default cooling time of 30 s.

**Figure 8:**
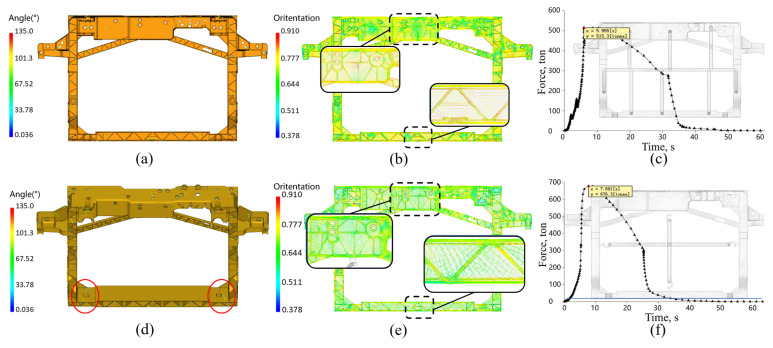
Simulation results using (**a**–**c**) sequential valve injection; (**d**–**f**) conventional simultaneous injection: (**a**,**d**) weld line distribution; (**b**,**e**) fiber orientation tensor; (**c**,**f**) injection pressure.

The injection molding simulation results for case 1 and case 2 are shown in Figure 8. Case 1, which utilizes sequential valve control, does not exhibit weld lines between the two gates where the melted fronts converge. In contrast, although case 2 employs fewer gates (offering cost advantages), it results in significant weld lines at the corners of the front-end module, which are critical drawbacks for load-bearing components. In terms of fiber orientation, case 1 demonstrates relatively uniform fiber alignment throughout the entire front-end module, with only minor discontinuities near the weld line regions, while case 2 shows pronounced fiber orientation discontinuities at weld line locations due to its gate arrangement and lack of sequential valve control. Consequently, compared to case 2, the gating system of case 1 yields a more favorable fiber orientation pattern for enhanced mechanical performance. Moreover, the maximum injection pressure for case 1 is 515.3 MPa, while case 2 reaches a significantly higher value of 676.3 MPa, which further indicates that the gating system of case 1 is more favorable.

**Figure 9:**
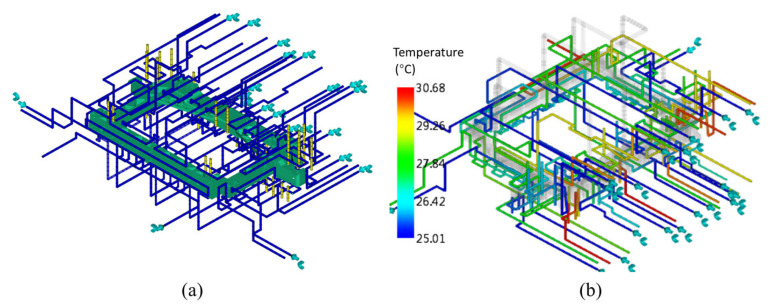
Cooling system scheme for the sequential valve injection (**a**) and the corresponding temperature distribution at the moment of maximum outlet-inlet temperature difference (**b**).

After comprehensive evaluation, the sequential valve control of case 1 is ultimately adopted, with well-defined time for filling and packing. However, the cooling stage—from the end of packing to mold opening—accounts for over 50% of the total cycle time. Therefore, to enhance production efficiency, it is essential to optimize the cooling system, especially the layout of cooling channels. The cooling channel design must holistically balance cooling efficiency, mold structural compatibility, and manufacturability. Without interfering with other component arrangements, the optimized cooling system shown in Figure 9a is optimally designed based on the front-end module structure and gating system, with the temperature distribution at the moment of the maximum inlet–outlet temperature difference shown in Figure 9b. Clearly, the maximum temperature difference is less than 5 °C, indicating that the temperature difference between the inlet and outlet remains stable within 5 °C throughout the entire cooling process, fully complying with cooling process specifications while delivering a superior cooling performance.

### 3.2. Optimization of Injection Molding Process

For design of experiment (DOE), the warpage of the front-end module is selected as evaluation criterion, with five key process parameters identified as influencing factors: mold temperature T, melt temperature $T_{m}$, injection time $t_{i}$, packing duration $t_{p}$, and packing pressure (expressed as a percentage of injection pressure) P. Among them, the setting ranges for T and $T_{m}$ strictly adhere to the recommended process window for PP + LGF40 GFRP (mold temperature: 20–60 °C, melt temperature: 200–260 °C) given by Moldflow software, while the ranges of the other parameters are shown in Table 4. The experiment is designed using an L_16_(4^5^) saturated orthogonal array, enabling comprehensive investigation of five factors at four levels, resulting in 16 distinct process parameter combinations, as listed in Table 5. Injection molding simulation with each process parameter combination is conducted with warpages listed in Table 5.

**Figure 10:**
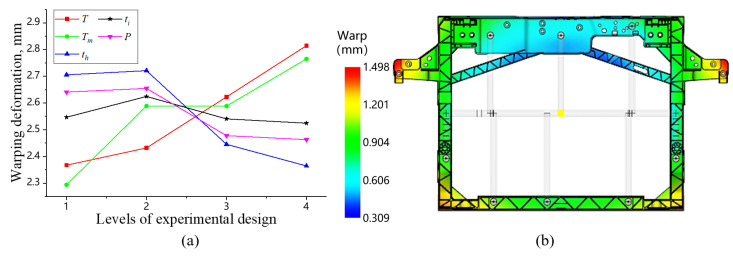
Effect of injection molding process on warpage trends (**a**) and warpage distribution under optimal process parameter combination (**b**).

Figure 10a shows the influence of process parameters on warpage. It can be found that $T_{m}$, T, and P are the primary factors affecting warpage, ranked in descending order as $T_{m}$ > T > P > $t_{i}$ > $t_{p}$. Under current experimental conditions, the optimal process parameter combination is determined to be T = 20 °C, $T_{m}$ = 210 °C, P = 90% $t_{i}$ = 6 s, and $t_{p}$ = 25 s. Simulation employing the optimized process parameter combination is conducted. The result shows that the warpage is significantly reduced to 1.498 mm. Compared with the average warpage from the 16 sets of orthogonal experiments, the optimal parameter combination represents a reduction of 41.5% in warpage, which can provide a reliable theoretical basis and technical guidance for injection molding process setting in actual production.

## 4. Performance Evaluation Mapping Injection Molding History

### 4.1. Mounting Point Stiffness Simulation

**Figure 11:**
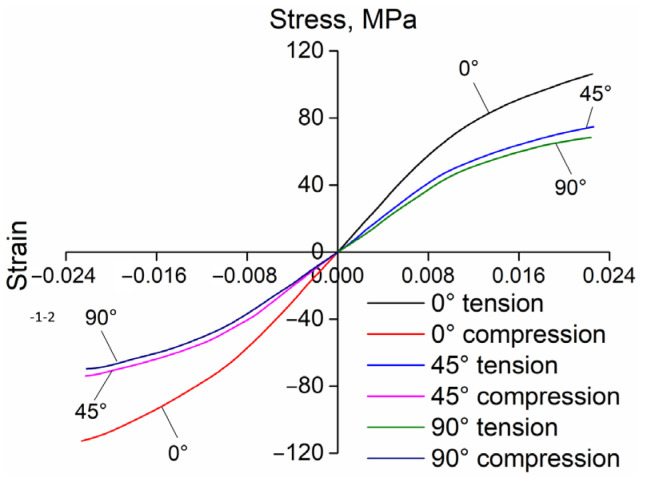
Ramberg–Osgood curves of PP + LGF40 GFRP.

For experimental verification, some front-end module samples are fabricated using the optimal process parameter combination. Tensile specimens are then extracted along three fiber orientations (0°, 45°, and 90°) with the following dimensions: width 19 mm, thickness 3 mm, gauge width 13 mm, transition radius 76 mm, gauge length 50 mm, parallel length 57 mm, and total specimen length 165 mm. Compression samples are also extracted in the above three directions with a diameter of 8 mm and a height of 12 mm. Quasi-static tension and compression are conducted at room temperature using an Instron 1342 universal testing machine with a strain rate of 0.001 s^−1^. The obtained stress–stain data are imported into Autodesk Helius PFA 2019 for characterization processing, yielding a set of Ramberg–Osgood curves [51]. The Ramberg–Osgood model is a theoretical mechanical model, which is used to describe the stress–strain relationship of materials near the yield point. It can be expressed as

(1) $$\varepsilon =\frac{\sigma }{E}+K{\left(\frac{\sigma }{E}\right)}^{k}$$

where ε is the total strain, σ is the total stress, E is the elastic modulus, and K and n are material-dependent constants. The Ramberg–Osgood curves for PP + LGF40 GFRP obtained through characterization are shown in Figure 11.

**Figure 12:**
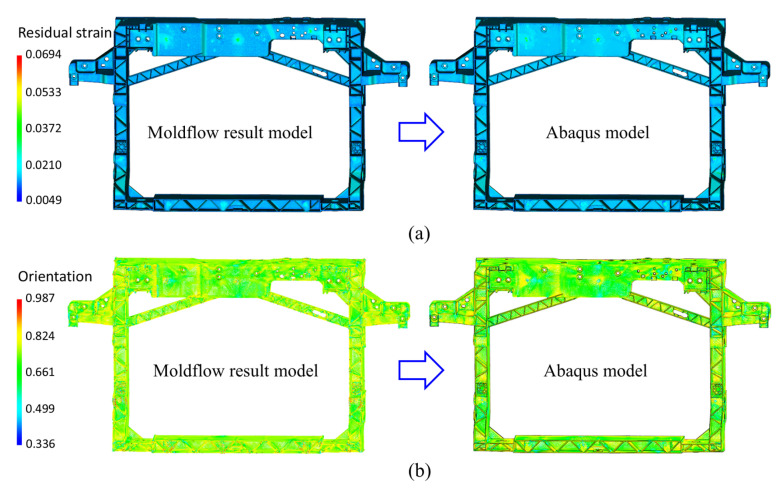
Results of mapping the (**a**) residual strain and (**b**) fiber orientation tensor to structural simulation models.

Firstly, the filling, packing, and warpage of the front-end module are simulated using Moldflow software based on the obtained Ramberg–Osgood curve, with resulting files then exported to Autodesk Helius PFA. The simulation outputs include five file formats: (1) SDY—computational input file containing model mesh, nodal data, boundary condition, processing setting, and material property; (2) LSP—resulting files storing layer-specific stress field, thermal property, and mechanical performance data; (3) OF1—complete filling simulation results excluding weld line and air entrapment; (4) OO1—fiber orientation distribution results; (5) WS3—weld interface data. Subsequently, the input and output files from Moldflow simulation are imported along with the stiffness simulation input file (.inp) from Abaqus into the Advanced Material Exchange module of Autodesk Helius PFA, while simultaneously aligning the Moldflow model with the Abaqus model. Finally, mapping the fiber orientation and residual stress–strain distribution onto the structural simulation model of Abaqus. The distributions of fiber orientation tensor and residual strain, as well as the corresponding Abaqus model after injection molding history mapping, are shown in Figure 12. In the mapped Abaqus model, each finite element is assigned a unique material property corresponding to the manufacturing-induced variations. This methodology bridges a relationship between process and service performance by maintaining traceability from injection molding parameters to mechanical behavior.

Based on the above model mapped with injection molding history, four simulation models of mounting point stiffness for the latch, headlight, horn and radiator are established, respectively, and the .inp files are exported and submitted to Abaqus for calculation. The maximum displacements of the measuring points are extracted and counted, as shown in Table 2. Clearly, the injection molding history has a significant effect on the performance evaluation.

### 4.2. Test Verification and Analysis

**Figure 13:**
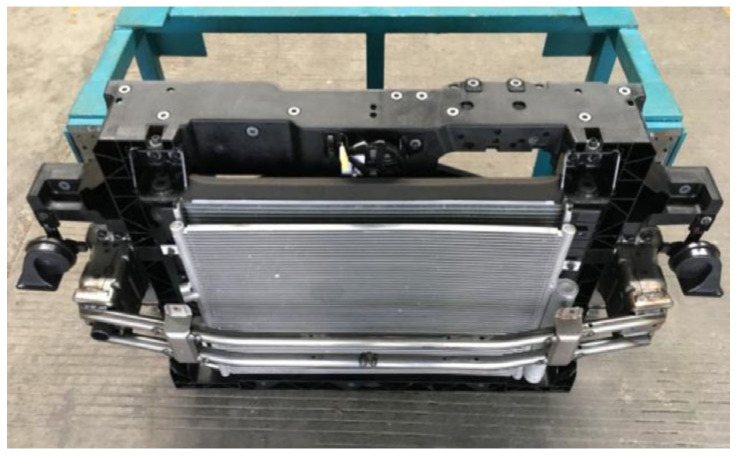
Mounting point stiffness test rig of the studied front-end module and its assembly state.

**Figure 14:**
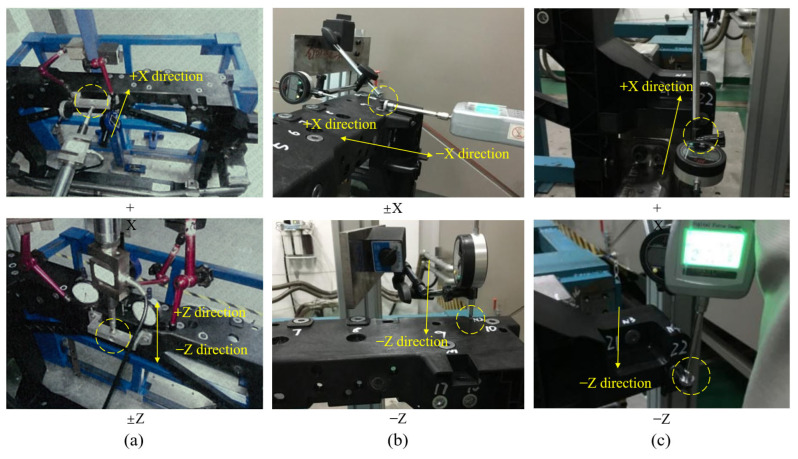
Mounting point stiffness tests for (**a**) latch, (**b**) headlight, (**c**) horn.

To validate the accuracy of performance evaluation for the studied front-end module with mapped injection molding history, stiffness tests are conducted on each mounting point, followed by an assessment of whether they meet the design requirements. Mounting point stiffness tests are conducted in accordance with the test specification from an automotive manufacturer. Before testing, the front-end module prototype is mounted on a test rig, with connection positions and methods consistent with the actual vehicle, as shown in Figure 13. All tests are conducted at an ambient temperature of 23 °C. The loading and measuring points for the mounting point stiffness test are shown in Figure 14, and the concentrated force loads applied to each mounting point are listed in Table 1.

It should be noted that the stiffness tests for the latch and radiator mounting points are conducted on an electric servo test system, while the headlight and horn mounting points are tested using a handheld force measurement device. Displacement measurements for all tests are performed using a digital displacement gauge. The digital displacement gauge is fixed on the test rig using a universal bracket to ensure it is unaffected by the loading process during testing. All loads are applied as concentrated forces, progressively increasing from 0 N to the designed values, maintained at peak for 10 s, with the maximum displacements recorded by the digital displacement gauge. The mounting point stiffness tests for the latch, headlight, and horn are illustrated in Figure 14, with the statistical results of the maximum displacement listed in Table 2.

The maximum displacement measured at the latch mounting point is 5.21 mm in the +X direction and 1.97 mm in the +Z or −Z direction, both values being below their specified control limits of 7.0 mm and 2.0 mm, respectively, indicating that the latch mounting point stiffness meets the design requirements. For the headlight mounting point, the maximum displacement is 0.37 mm in the +X direction and 0.21 mm in the −Z direction, both of which are below the control limit of 1.0 mm. Therefore, the headlight mounting point stiffness also complies with the design requirements. In addition, the other installation points also meet the design requirements.

### 4.3. Error Analysis of Simulation and Test

**Figure 15:**
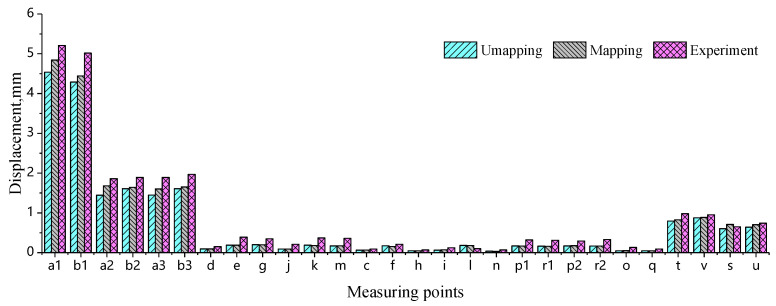
Comparison between simulated and experimental displacements at each measurement point under different conditions.

For comparative purposes, simulations utilizing isotropic material model without mapping the injection molding history are conducted. The results reveal that two distinct modeling approaches result in significant divergence. Figure 15 shows a statistical comparison of the maximum displacements obtained from simulations without injection molding history mapping, simulations with injection molding history mapping, and experimental tests under different loading conditions. Compared to the experimental results, the simulated maximum displacements for all mounting point stiffness values are smaller. Notably, the simulated maximum displacements without injection molding history mapping are lower than those obtained with injection molding history mapping. That is, the simulation results accounting for the fiber orientation and residual stress–strain field are relatively larger. Moreover, this trend becomes more pronounced as the deformation displacement increases.

To better analyze the differences between them, the commonly used engineering metric of percentage error $E_{r}$ is employed to quantify the discrepancy between simulation and test results. The expression is as follows:

(2) $$E_{r}=\frac{\left|b-b_{t}\right|}{b_{t}}\times 100\%$$

where b is the simulation result and $b_{t}$ is the experimentally measured value. Although the experimental measurements are conducted in accordance with the test specification from an automotive manufacturer, unavoidable errors are introduced by factors such as the loading method, handheld push–pull force gauge, and digital displacement gauge. Additionally, the loading points and measurement points in the experiments cannot perfectly align with those in the simulations, which also introduces discrepancies. Therefore, the comparison between simulation and experimental results should be analyzed within the scope of these inherent errors.

**Figure 16:**
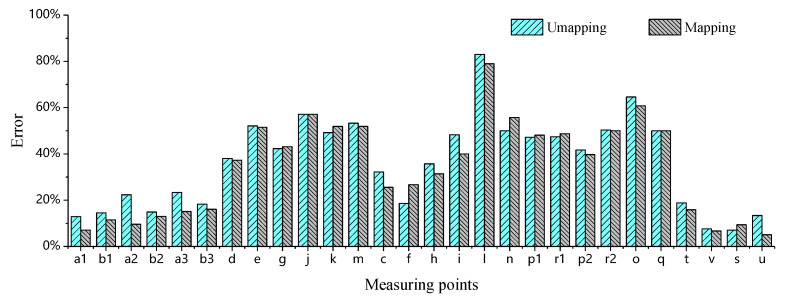
Percentage deviation between simulation and experiment at each measurement point under different conditions.

**Figure 17:**
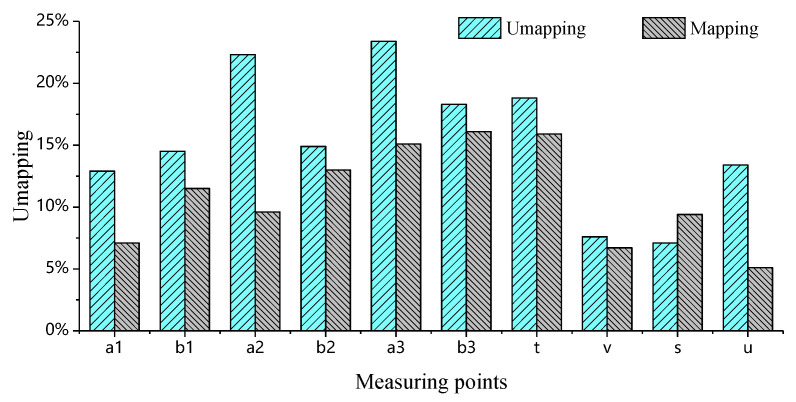
Percentage deviation between simulation and experiment results at specific measurement points.

The errors between simulations (both with and without injection molding history mapping) and measurements are calculated using Equation (2), with the statistical results shown in Figure 16. It can be observed that when the experimentally measured displacements are small, both simulation approaches exhibit significant deviations from the test results, with maximum $E_{r}$ reaching 83% and 79%, respectively. It is particularly noteworthy that when the control limit at the mounting points is below 0.40 mm, the corresponding average percentage errors $E_{r}$ reach 47.9% and 48.0%, respectively. Such large margins of error render these measurements unreliable for reference purposes. These demonstrate that, under the inherent presence of systematic errors, when experimental measurements are sufficiently small, the discrepancies between simulation and test results remain significant regardless of whether the injection molding history is mapped or not. From an engineering standpoint, these values are negligible in magnitude and maintain substantial margins from evaluation thresholds, thus failing to qualify as valid indicators for assessing simulation accuracy. However, when the measured displacement exceeds 0.65 mm, the percentage errors $E_{r}$ between the experimental results and both unmapped and injection-molding-history-mapped simulations decrease significantly, as illustrated in Figure 17. Here, the maximum $E_{r}$ values are 23.49% (unmapped) and 19.14% (mapped), while the minimum $E_{r}$ dropped to 7.08% and 5.14%, respectively. The average $E_{r}$ further decreases from 15.36% (unmapped) to 11.78% (mapped).

Based on the above analysis, it can be concluded that simulations with mapped injection molding history consistently yield smaller percentage errors $E_{r}$ compared to those without molding history mapping, validating the importance of accounting for fiber orientation and residual stress–strain in accuracy-critical scenarios.

To more intuitively evaluate the superiority of the mapped injection molding history method, the range R and variance $s^{2}$ are introduced to quantify the deviation from the mean error. The expressions for R and $s^{2}$ are as follows:

(3) $$R=X_{max}-X_{min}$$

(4) $$s^{2}=\frac{\sum_{i=1}^{n}{\left(x_{i}-x\right)}^{2}}{n}$$

(5) $$x=\frac{\sum_{i=1}^{n}x_{i}}{n}$$

where $X_{max}$ is the maximum value, $X_{min}$ is the minimum value, $x_{i}$ is the *i*-th sample, x is the mean of the sample array, and n is the number of samples. The calculated R of the simulation errors is reduced from 16.41% (without history mapping) to 14.00% (with history mapping), while the variance $s^{2}$ decreases from 0.00304 to 0.002462. These quantitative improvements confirm that the mapped injection molding history method achieves significantly lower error dispersion, as evidenced by both smaller R and $s^{2}$. The superior performance of the mapped injection molding history method is fully demonstrated, which can provide more reliable and consistent results for engineering applications requiring high precision.

## 5. Conclusions

1. Under ±Z-directional loading of 1000 N, the initial design of the latch mounting point exhibits a displacement of 2.254 mm, exceeding the regulatory limit (<2.0 mm). After topology optimization, the displacement is reduced to 1.609 mm. Experimental validation confirms that simulations mapping injection molding data (fiber orientation, residual stress–strain) yield results closer to the measured values, and the anisotropic model demonstrates significantly lower errors than the isotropic model.
2. Case 1, which utilizes sequential valve gate control, demonstrates superior performance in both weld line quality and fiber orientation control compared to conventional gating systems. Through orthogonal experiment, the optimal process parameter combination is determined—mold temperature of 20 °C, melt temperature of 210 °C, packing pressure with 90% of injection pressure, injection time of 6 s, and packing time of 25 s—which reduces the warpage to 1.498 mm, with a 41.5% reduction compared to the average warpage obtained from the orthogonal experiment.
3. The displacement results obtained from the simulation based on mapped injection molding historical data are closer to the experimental values, with the error decreasing as the displacement increases. When the measured displacement exceeds 0.65 mm, the simulation using mapped data demonstrates superior performance in terms of percentage error $E_{r}$, range R, and variance $s^{2}$, validating the engineering applicability of the anisotropic simulation.
4. When the tested displacement is small (<0.65 mm), significant deviations exist between simulations and measurements regardless of whether injection molding historical data is mapped, which is primarily due to systematic errors in experimental equipment and operations. Such errors are inherently unavoidable in engineering practice, necessitating reliance on large-displacement condition data as the primary basis for optimization.

## Figures and Tables

**Table 1:** Main mechanical control limits for front-end modules of vehicle.

Test Item	Load	Displacement
Latch mounting points	+X 1000 N	≤7 mm
	+Z 1000 N	≤2 mm
	−Z 1000 N	≤2 mm
Headlight mounting points	+X 100 N	≤1 mm
	−Z 100 N	≤1 mm
Radiator mounting points	+X 600 N	≤1 mm
	−X 600 N	≤1 mm
	−Z 600 N	≤1 mm
Horn mounting points	+X 300 N	≤2 mm
	−Z 300 N	≤2 mm

**Table 2:** Simulation and test results of the mounting point stiffness.

Analysis Item	Load/N	Test Point	Initial Design/mm	Optimize Case/mm		
				Unmapping	Mapping	Test
Latch mounting points	+X 1000	a_1_	5.263	4.536	4.842	5.21
		b_1_	5.030	4.290	4.443	5.02
	+Z 1000	a_2_	2.008	1.446	1.682	1.86
		b_2_	2.254	1.609	1.644	1.89
	−Z 1000	a_3_	2.008	1.447	1.604	1.89
		b_3_	2.254	1.609	1.653	1.97
Headlight mounting points	+X 100	d	0.098	0.093	0.094	0.15
		e	0.196	0.187	0.189	0.39
		g	0.209	0.202	0.199	0.35
		j	0.093	0.090	0.090	0.21
		k	0.191	0.188	0.178	0.37
		m	0.172	0.168	0.173	0.36
	−Z 100	c	0.074	0.061	0.067	0.09
		f	0.182	0.171	0.154	0.21
		h	0.036	0.045	0.048	0.07
		i	0.067	0.062	0.072	0.12
		l	0.170	0.183	0.179	0.10
		n	0.041	0.035	0.031	0.07
Radiator mounting points	+X 600	p_1_	0.167	0.169	0.166	0.32
		r_1_	0.163	0.163	0.159	0.31
	-X 600	p_2_	0.167	0.169	0.175	0.29
		r_2_	0.163	0.164	0.165	0.33
	−Z 600	o	0.046	0.046	0.051	0.13
		q	0.046	0.045	0.045	0.09
Horn mounting points	+X 300	t	0.832	0.796	0.824	0.98
		v	0.853	0.878	0.886	0.95
	−Z 300	s	0.623	0.604	0.711	0.65
		u	0.647	0.641	0.702	0.74

**Table 3:** Basic mechanical properties of PP + LGF40 GFRP.

Property	Value
Modulus of elasticity in the first direction *E*_1_	7266.9 MPa
Modulus of elasticity in the second direction *E*_2_	4488.6 MPa
Poisson’s ratio *v*_12_	0.338
Poisson’s ratio *v*_22_	0.436
Shear modulus *G*_12_	1621 MPa

**Table 4:** DOE factors and levels for injection molding.

Factors	T (°C)	$T_{m}$ (°C)	$t_{p}$ (s)	$t_{i}$ (s)	P (%)
Level 1	20	210	10	4.5	60
Level 2	30	220	15	5.0	70
Level 3	40	230	20	5.5	80
Level 4	50	240	25	6.0	90

**Table 5:** Sixteen sets of DOE injection molding process parameter combinations and their corresponding warpage simulation results.

Number	T (°C)	$T_{m}$ (°C)	$t_{p}$ (s)	$t_{i}$ (s)	P (%)	Warpage (mm)
1	20	210	10	4.5	60	2.318
2	20	220	15	5.0	70	2.720
3	20	230	20	5.5	80	2.183
4	20	240	25	6.0	90	2.248
5	30	210	15	5.5	90	2.214
6	30	220	10	6.0	80	2.492
7	30	230	25	4.5	70	2.350
8	30	240	20	5.0	60	2.671
9	40	210	20	6.0	70	2.305
10	40	220	25	5.5	60	2.521
11	40	230	10	5.0	90	2.768
12	40	240	15	4.5	80	2.897
13	50	210	25	5.0	80	2.339
14	50	220	20	4.5	90	2.622
15	50	230	15	6.0	60	3.053
16	50	240	10	5.5	70	3.244

## Data Availability

The original contributions presented in this study are included in the article. Further inquiries can be directed to the corresponding author.
